# Microneedle Coating Techniques for Transdermal Drug Delivery

**DOI:** 10.3390/pharmaceutics7040486

**Published:** 2015-11-05

**Authors:** Rita Haj-Ahmad, Hashim Khan, Muhammad Sohail Arshad, Manoochehr Rasekh, Amjad Hussain, Susannah Walsh, Xiang Li, Ming-Wei Chang, Zeeshan Ahmad

**Affiliations:** 1 School of Pharmacy, De Montfort University, Leicester LE1 9BH, UK; E-Mails: rita.haj-ahmad@sunderland.ac.uk (R.H.-A.); hashimkhan15@googlemail.com (H.K.); sohail_arshad79@yahoo.com (M.S.A.); m.rasekh@alumni.ucl.ac.uk (M.R.); amjad_husein@hotmail.com (A.H.); SWalsh@dmu.ac.uk (S.W.);; 2 Department of Pharmacy, Bahauddin Zakariya University, Multan 60800, Pakistan; 3 College of Pharmacy, University of the Punjab, Lahore 54000, Pakistan; 4 State Key Laboratory of Silicon Materials, School of Materials Science and Engineering, Zhejiang University, Hangzhou 310027, China; E-Mail: xiang.li@zju.edu.cn; 5 College of Biomedical Engineering and Instrument Science, Zhejiang University, Hangzhou 310027, China; 6 Zhejiang Provincial Key Laboratory of Cardio-Cerebral Vascular Detection Technology and Medicinal Effectiveness Appraisal, Zhejiang University, Hangzhou 310027, China

**Keywords:** microneedles, coatings, drug delivery, coating process, films, particles

## Abstract

Drug administration via the transdermal route is an evolving field that provides an alternative to oral and parenteral routes of therapy. Several microneedle (MN) based approaches have been developed. Among these, coated MNs (typically where drug is deposited on MN tips) are a minimally invasive method to deliver drugs and vaccines through the skin. In this review, we describe several processes to coat MNs. These include dip coating, gas jet drying, spray coating, electrohydrodynamic atomisation (EHDA) based processes and piezoelectric inkjet printing. Examples of process mechanisms, conditions and tested formulations are provided. As these processes are independent techniques, modifications to facilitate MN coatings are elucidated. In summary, the outcomes and potential value for each technique provides opportunities to overcome formulation or dosage form limitations. While there are significant developments in solid degradable MNs, coated MNs (through the various techniques described) have potential to be utilized in personalized drug delivery via controlled deposition onto MN templates.

## 1. Introduction

Transdermal drug delivery (e.g., transdermal patches) offers an attractive alternative to oral and parenteral routes of drug administration. These methods have provided significant contributions towards pharmaceutical (emerging therapies) applications (e.g., vaccination, skin treatment and for controlled release). Such administration routes have a distinct advantage in overcoming the first pass effect of the liver, which can prematurely metabolize active drugs [1]. Most transdermal systems are inexpensive, non-invasive, are self-administrated and provide sustained release of the active drug (up to one week) which improves patient compliance [1,2]. However, transdermal delivery is limited to a number of drugs due to the major barrier function of the skin [3]. The skin has three layers: epidermis (which is the main physical barrier), dermis and a fat layer. The epidermis of the skin has five separate layers: stratum basale (basal or also known as the germinativum cell layer), stratum spinosum (spinous or also known as the prickle cell layer), stratum granulosum (granular cell layer), stratum corneum (also termed the horny layer) and stratum lucidum (located between the stratum granulosum and stratum corneum). Stratum corneum (the superficial layer of the skin) is the most challenging barrier for transdermal drug delivery [4,5].

Several enhancement approaches have been utilized in order to increase skin permeability, ranging from chemical/lipid penetration enhancers to non-cavitation ultrasound, thermal ablation, iontophoresis, sonophoresis, microdermabrasion, electroporation, cavitational ultrasound and microneedles (MNs) [1,3,6,7,8,9,10]. Although the principles and mechanisms of these approaches are different, these methods share the same aim of enhancing the movement of the drug through the stratum corneum, either through pore formation or improved diffusive interaction. This facilitates the movement of drug molecules towards the blood supply in the skin [8] or the Langerhans cells for vaccine delivery.

In the last twenty years, MNs have been proposed as pain-free systems (significantly less painful than a 26-gauge hypodermic needle) [11] with high potential of transdermal drug delivery (via perforated regions of the stratum corneum). This is achieved by avoiding or minimizing underlying pain nerve stimulation [5]. MNs have been manufactured in different shapes and sizes from a broad range of materials to deliver drugs with variable molecular size and weight [10,12]. MNs present an attractive drug delivery approach with the potential for delivering molecules with functional properties and also macromolecules [9], e.g., bovine serum albumin [6,8], calcein [13], desmopressin [14], parathyroid hormone PTH [15], insulin [16], OVA protein [17] and horseradish peroxidase [18]. Vaccines that have been coated onto MNs include hepatitis B antigens [19], inactivated influenza virus [20] and virus-like particles (influenza) [21].

MNs have the advantages of delivering small quantities of high-potency medication through the skin to minimize the pain factor [22] and allowing precise tissue localization for drug delivery [13]. Moreover, large active pharmaceutical molecules can be administered without causing pain using MNs as they only puncture the epidermal skin layer [8]. They can also be used for biological analysis (via skin blood contact). Due to minimal invasiveness they offer the advantage of fast healing at the injection site (local skin area) with low risk of microbial infection [3]. MNs also have the added benefit of rapid penetration of drugs directly into the blood circulation system (compared to skin diffusive approaches), subsequently avoiding the first pass effect of the liver and the digestive enzymes of the gastrointestinal tract [1]. Furthermore, a selection of active and functional molecules (small molecules, e.g., calcein, and large molecules, e.g., proteins) and vaccines are well tolerated through controlled MN delivery [3]. While this technique is termed pain-free, it is also minimally invasive without long-term oedema or erythema. A rapid onset of drug delivery can be accomplished by coupling MNs with an electrically controlled micro-pump which can effectively determine the rate of drug delivery as compared with other drug delivery approaches [23].

The application of MNs is limited due to their size and material properties. MN tips are prone to breakage and subsequent embedment within the skin if prepared from materials with poor mechanical properties [7,23]. Furthermore, the thickness of the stratum corneum and other skin layers differs between specimen and anatomical location. Accordingly, the penetration depth of the drug is variable and dosage accuracy is limited. Allergy prone or sensitive skin is also affected by MNs (e.g., material type and skin irritation). Improper MN application technique can also lead to skin inflammation by increased drug impregnation under the skin [3].

This short review focuses on MNs as transdermal drug delivery systems. The review provides an overview of the advantages and disadvantages of MN systems. Moreover, examples of different MN structures, coating methods and coating formulations are highlighted. Specifically, emerging and existing MN coating methods are summarized in a table (Table 1) that details key aspects for these developments (e.g., drug, coating materials, processes and main outcomes).

## 2. Microneedle Mechanism and Design

MNs have been developed in ways that enable them to share advantages of both hypodermic needles and transdermal patches to deliver drugs through the skin at therapeutically desirable quantities [6,7,8,9,10]. The combinatorial design of such MNs has overcome the limitations of the hypodermic needles (pain and risk associated) and transdermal patches (limited by the transport barrier provided by stratum corneum) [11].

MNs are significantly different from hypodermic needles based on their length and the pore size they generate (ranging from sub-microns to millimeters). Generally, MN patches or substrates possess similar basic design elements such as an ordered array of MNs ranging from a few to a few hundred in number. MNs are prepared from various materials and manufactured in a plethora of shapes and sizes [6,7,8,9,10,11]. They were originally micro-fabricated from silicon and later manufactured from metals, polymers and ceramics (including glass).

The design of MNs and the way in which the drug or formulation is incorporated to target the skin varies. There are four main MN designs. The first is an array of solid MNs (with no drug) which is used to penetrate into the skin, increasing skin permeability for the intended drug (e.g., drug usually applied using patch or topical formulation). The second type is degradable MNs, prepared using biodegradable polymers (e.g., poly(lactic-*co*-glycolic acid) (PLGA) polymer). These degrade safely in the skin while providing a sustained release of drug [24,25]. This one-step method has provided precise drug dosing with 90% bioavailability [26]. However, low bioavailabilites (32%) have been reported for peptide leuprolide acetate (1.2 kDa) which has shown metabolic instability in the skin [27]. The third type is injectable hollow MNs which permit the continuous delivery of drug into the skin [13]. MNs contain hollow bores which minimize invasiveness and reduce pain typically experienced during hypodermic needle drug delivery. Hollow MNs are inserted into the skin, after which liquid formulation is actively or passively infused into the injected tissue. The final type is coated MNs, in which design (conventionally) metallic MNs are coated with a drug formulation. These MNs are ideal for the delivery of potent drugs into the skin after piercing through the stratum corneum [3]. For instance, vaccines coated onto MNs can endorse their desired response by interacting with the dermal dendritic cells (Langerhans cells) [28,29]. Moreover, high molecular weight molecules can be rapidly delivered into the skin, through self-implementation, such as with “Band-Aid”-like systems [13]. Coated MNs also possess the advantage of prolonged shelf-life. For example, 98% integrity of desmopressin (synthetic peptide hormone) coated onto MNs was maintained after 6 months of storage under nitrogen at ambient conditions [14]. In general, the coating process, excipients, selected active drug and formulation all have an impact on MN coatings. High drug loading volumes with improved strength, protection against moisture and controlled drug release are other factors which provide benefits of coated MN systems [13]. It is also imperative to have a coating thickness in the micron range which does not occlude the needles or inhibit skin penetration. However, the quantity of the drug to be administered using this method is limited to the amount of the drug that can be coated onto the tips and shafts of MNs. This is normally less than 1 mg for small MNs arrays [13]. Coated MNs retain their mechanical strength; however, their tip sharpness is reduced and this impacts skin penetration ability.

Gill *et al.*, investigated the influence of MN geometry (using MNs with variations in length, thickness, width, tip angle and number of MNs on a patch) on pain compared to a 26-gage hypodermal needle. All MNs investigated were 5% to 40% less painful than the selected hypodermic needle. The thickness, width and tip angle had no significant impact on pain. However, decreasing the number and length of the MNs decreased the pain significantly. In addition, increasing the number of MNs 10-fold resulted in a two-fold increase in pain (by score). They also concluded that a three-fold increase in MN length resulted in a seven-fold increase in pain (by score) [11].

## 3. Microneedle Coating Methods

**Figure 1:**
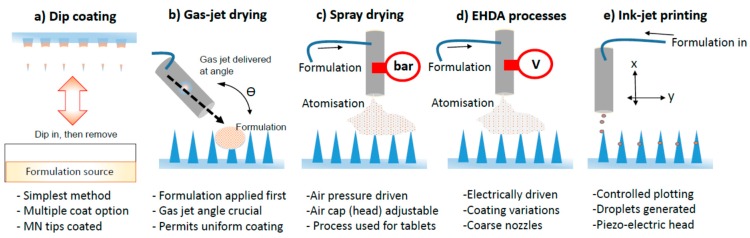
Illustrated examples of techniques used to coat MNs. (**a**) Dip coating; (**b**) Gas-jet drying; (**c**) Spray drying; (**d**) EHDA processes; (**e**) Ink-jet printing.

Coated MNs are attractive devices for drug delivery through the skin. Previously, MNs were coated by immersing patches in a liquid solution for several hours to ensure a full coat onto their surface. However, this simple procedure has the drawback of drug wastage and loss, variable coating thickness of active onto MNs and thus inaccuracy in drug dosage [13]. Different coating approaches have been developed to date, including dip coating, gas-jet drying, spray-coating, EHDA based processes and piezoelectric ink-jet printing. These are illustrated in Figure 1.

### 3.1. Dip Coating

The dip coating process is the simplest procedure to coat MNs. MNs are first dipped into the formulation and then withdrawn. This step produces a liquid film on MNs. The liquid layer is then allowed to dry to form a solid film coating. The dip coating method has been utilized to deliver hydrophilic and hydrophobic drugs. Several biomolecules have been coated onto MNs using this technique, e.g., proteins, viruses and DNA, for rapid transdermal delivery [30].

Ma and Gill, have reported one of the earliest attempts at coating a hydrophobic drug onto MNs using molten dip coating. Lidocaine (*M*_W_ = 270.8 Da) is a hydrophobic drug which is used as a local anesthetic agent. Usually, the administration of lidocaine to the patient is either topically (*i.e.*, cream) or parentally (*i.e.*, injection). Here, the molten dip coating process was used to develop uniform lidocaine coated MNs for transdermal delivery. Polyethylene glycol (PEG) was used as a hydrophilic matrix with a lidocaine base to create the solid dispersion. Drug stability was achieved even at elevated temperatures of ~130 °C. The mass fraction of lidocaine in the drug dispersion had an impact on the PEG-lidocaine molten solution, as decreasing the mass fraction of lidocaine increased the solution viscosity. Compared to the conventional 1 h application of topical cream (0.15 g EMLA^®^, a 5% emulsion of equal quantities of 2.5% lidocaine and 2.5% prilocaine), the *in vitro* dissolution studies of PEG-lidocaine coated MNs in porcine skin demonstrated a significant increase in lidocaine delivery within 3 min [31].

Human growth hormone (191 amino acids) is a peptide which is important for growth, cell regeneration and reproduction in humans. Recombinant human growth hormone (rhGH) was successfully dip coated onto titanium MNs for transdermal delivery (200 mg/mL; 20% *w*/*w*). rhGH MN patches were stable for 6 months at 40 °C. Compared to commercial subcutaneous Norditropin injection (rhGH), rhGH coated MNs were found to provide a similar absolute bioavailability. This suggests rhGH MN patches have potential to replace rhGH injections in the pharmaceutical market due to reduced pain benefits and ease of administration [32].

The dip coating method works by submerging MNs into a drug solution and could result in a non-uniform coating [30]. Surface tension is a dominant feature which prevents uniform MN coatings as they are closely spaced [33]. Gill and Prausnitz developed a micron scale dip coating process to produce uniformly coated MNs. This process relies on coating MN shafts with a thick layer of coating and not the base substrate using a highly viscose formulation to decrease surface tension. MNs of different geometries and configurations (single MNs, in-plane rows of MNs, and out-of-plane arrays of MNs) were laser cut from stainless steel sheets using an infrared laser followed by electropolishing. This dip coating design used dip holes with similar dimensions of MNs rather than a large open coating surfaces to avoid rising of the meniscus subsequently masking the base substrate within the spaced MNs. MN shafts were coated with very small volumes of formulation, ranging from 10 μL for single MNs to 100 μL for 50 MNs (out-of-plane arrays). A variety of biomolecules (3% Vitamin B, 1% bovine serum albumin, 0.05% g Wiz™ luciferase plasmid DNA, 0.01% suforhodamine and modified vaccinia virus) were utilized with a modified coating solution (1% (*w*/*v*) of low viscosity carboxymethyl cellulose sodium salt and 0.5% (*w*/*v*) Lutrol F-68 NF). Here, MN shafts were coated without contaminating the base. Coated materials dissolved (to liquid form) within 20 s in porcine cadaver skin with complete delivery into the skin [13].

### 3.2. Gas Jet Drying

The slow drying process associated with the dip coating approach is limited practically, especially for curved MNs. While the drug coating solution is still wet on the MN surface, the solution has potential to move (gravitational and low surface tension spreading) and relocate off the MN surface, reducing and varying the desired dose. Even the multi-dip coating approach is potentially problematic as a thick multi-layer [34] coating (of solution) accumulates and dries at the base substrate. The gas jet drying approach was developed by Chen *et al.*, to overcome the addressed problem, especially for very small (<90 micron length) and very closely (~20.000 cm^−2^) spaced MNs. Solid silicon microprojections were sputter coated with a thin layer of gold. The whole length of the microprojections was coated with a (6–8 µL) solution which possessed ideal surface tension and viscosity properties. The coating solution contained methylcellulose (works by increasing the viscosity and decreasing the surface tension of the coating solution at the same time), Quil-A (has benefits of serving as a surfactant to reduce the surface tension and works as a vaccine immune-stimulatory adjuvant) and selected concentrations of model active drugs (vaccines or fluorescent dyes). Formulations were applied and were modified using (6–8 m/s) a gas jet. The viscosity of the coated layer (5 µm thick) onto the microprojections increased rapidly, allowing the coated material to dry rather than relocate on the base substrate. This was followed by a fast gas jet (10 m/s) at an incident angle of 20° to remove all excess coating solution. The uniformly dried coating remained intact during skin penetration and the model drugs were released in 3 min within wet skin [17].

Vaccine delivery using MNs has merits over diffusion (the movement of drug from an area of high drug concentration to low concentration) delivery, biolistic MN delivery and electroporation delivery approaches. Vaccines comprise large molecular weight actives which has limited their MN based deployment. Typically, transdermal delivery favors small drug molecules (<500 Da) to pass through the stratum corneum. Chen *et al.*, have improved vaccine delivery efficiency by using gas jet coated MNs, by focusing on depositing actives on MN tips rather than whole MNs. This was performed by increasing the gas jet incident angle to 70°, removing the patch edge and rotating the patches during the coating process to ensure uniformity. The delivery efficiency of vaccines was increased from 7.3% ± 1.1% to 17.8% ± 1.5% (for incident angle 20°) simply by removing the patch edge. Delivery efficiency increased from 17.8% ± 1.5% (for incident angle 20°) to 32.5% ± 3.9% (for incident angle 70°) based on the incident angle with continuous rotating of patches [35].

### 3.3. Spray Coating

The spray coating process is similar to conventional coating approaches (e.g., used for coating tablets) to achieve millimetre thicknesses. The micron sized design of MNs (typically ~60 to 700 μm in height) requires a coating thickness in and below the micron range (particles size < 280 μm). Spray coating of microparticles onto MNs undergoes three steps. Firstly atomization, which is the generation of formulated microdroplets from the spray coater. Secondly, the deposition and adherence of droplets onto the surface of MNs. Finally, the coalescence of droplets on the substrate to form an intact film coating [36].

The spray coating process was first developed by McGrath *et al.* [36]. The configuration of the spraying system involved a 0.5 mm spraying nozzle which was connected to a compressed air pump and a coating solution. Silicon MNs were secured on the platform stage under the nozzle using double sided tape. Using a peristaltic pump or a syringe driver, the coating solution was injected into the nozzle for atomisation. Optimization of spray process parameters (e.g., atomization air pressure, gun-to-surface distance and air cap setting) are necessary for uniform film coating on the MN substrate. The film coat formation was highly affected by the coating solutions physio–chemical properties and the spray process parameters. Two coating materials were investigated (hydroxypropylmethylcellulose (HPMC) and carboxymethylcellulose (CMC)). HPMC was considered as a good film forming polymer that readily formed a film coat after optimizing process parameters. 1% *w*/*v* CMC solution had a higher surface tension than 5% *w*/*v* CMC and both HPMC solutions (5% and 12% *w*/*v*). Solutions with high polymer concentrations (5% *w*/*v* CMC, 12% *w*/*v* HPMC) demonstrated greater viscosities (compared to 1% *w*/*v* CMC, 5% *w*/*v* HPMC). These parameters adversely affected film-coating coalescence as the resulting film had a combination of small patches of coating and large uncoated areas. The addition of a surfactant (Tween 80) in the case of CMC solutions was necessary to assist coalescence of the sprayed droplets onto the silicon surface [36].

Spray coated solid MN patches were also used for transcutaneous delivery of live recombinant adenovirus (rADV) and modified vaccinia virus Ankara (MVA) vectors as vaccines. Viruses with suitable sugar-based formulations were spray coated in a dry form at the shaft of each silicon MN rather than the inter-needle space. A uniform coating pattern with an effective preservation of the virus’s activity was successfully delivered into the skin. The potency of recombinant virus vaccine coated onto MNs patches to induce antibody response, transcutaneous infection and induced antibody (or CD8+ T cell) response was equivalent to the response induced by transdermal injection of the same vaccine [37].

### 3.4. EHDA Based Processes

The electrohydrodynamic atomisation (EHDA) process has been developed to generate near uniform micro- and nano-meter scaled architectures in one step. The principle process was described by Grace and Marijnissen (1994). Here, atomized droplets are produced by an electrically imposed moving liquid (the electrical field generates charge inside droplets) that jet through a capillary nozzle exit and are subsequently collected over a ground electrode positioned below the nozzle tip [38]. The liquid used is a polymeric solution, or formulation, containing three main components (a solvent, polymer and active drug) and possibly other excipients. This technique has been widely used by researchers for various drug delivery therapies e.g., insulin [39], folic acid [40], titanium dioxide antimicrobial agent [41], gold used in gene delivery [42] *etc.* The EHDA system can generate both particles (electrospraying) and fibers (electrospinning).

Using the EHDA process, controlled particle and fibre coating thickness is achievable. Khan *et al.*, presented a multi-structural MN coating approach employing EHDA principles. Stainless steel MNs (600–900 µm height) were attached to the ground electrode. The coating solution (containing 5 wt % of selectable molecular weights of polyvinylpyrrolidone (PVP) dissolved in methanol:ethanol (50:50) and fluorescein dye (serving as a potential drug)) was infused into a three needle co-axial device which was connected to a high voltage supply. The MNs were coated with particles (100 nm to 3 µm) and fibres (400 nm to 1 µm) under controlled processing parameters, flow rate (~5–15 µL/min) and applied voltage (6–19 kV) at room temperature. This coating process can assist in advancing transdermal drug delivery [34]. This is especially important for sensitive biomolecules such as peptides and protein drugs which are stable during EHDA processes but unstable for delivery via oral route of administration [34,39,43]. In comparison to dip coating, the electrical spray-coating system (e.g., EHDA system) can be optimized to coat MN tips only and avoid coating the base substrate (by using surface insulating polymeric masks).

There are three main types of EHDA processes: single needle (in which the formulation is injected into a single nozzle by a single precision syringe pump), coaxial EHDA (this system uses two or more immiscible liquids which are fed through separately enveloped nozzles) and multiplexed EHDA (liquid formulation fed through a single or coaxial nozzle array). One of the benefits of EHDA using a coaxial system is protection of the sensitive drug from direct exposure to the biological environment [44]. This is related to the encapsulating architecture of the produced particle/fibres in which the biomolecule will be located in the core while the polymer forms the outer layer. Accordingly, the coaxial EHDA system is preferred as it can produce therapeutic particles with sustained and controlled release.

Certain parameters need to be considered when using EHDA process: flow rate, applied voltage and the distance between the nozzle and the collecting platform [45]. These factors have a high impact on the controlled particle size, size distribution, porosity, shape and surface charge. Applied voltage is the most important factor in controlling the jet mode and the size of resulting particles/fibres, as increasing the applied voltage will result in smaller particles/fibres [38]. Moreover, as the applied voltage is increased significantly the morphology of particles have the potential to transition from spherical to elongated (change in aspect ratio). An optimum voltage is determined to overcome the surface tension on the initially atomized droplets. Flow rate has a direct relation to the particle size and the size distribution of the produced architectures as the size reduces with decreasing flow rate. The distance between the tip of the needle and the ground platform has an impact on resulting morphologies. This is due to the relaxation time required for solidifying droplets to reach the deposition substrate, which can be increased by increasing the distance between the two points [38,46].

In addition to processing parameters, material properties also impact resulting particles/fibres as they significantly affect the jet stability. The viscosity, surface tension, electrical conductivity and density are all major factors to be considered before processing [47]. The most important parameter is the electric conductivity of the vehicle (solvent), as liquid with low electrical conductivity (e.g., heptane) cannot be used for single needle EHDA systems. The addition of antistatic additives or their coupling with an electrically conductive liquid improves process ability (e.g., through coaxial EHDA) [48]. Atomised particle size is directly related to solution viscosity and surface tension, while the reverse is true with regard to liquid density [38].

### 3.5. Piezoelectric Inkjet Printing

The inkjet printing approach is a valuable engineering apparatus that enables controlled distribution and accurate arrangement of fine liquid droplets (1–100 picoliters) onto a substrate (e.g., MNs) before solidification [49,50]. Unlike the dip coating method, inkjet printing technology requires formulations with low viscosity to avoid blockage of the jetting nozzle (which possess small dimensions) for a continuous MN coating process [51]. The concept involves dissolving selected excipients in a liquid to form an ink. The mechanism of drop formation and ejecting from the nozzle occurs by either (1) inducing vibrations on the material by using a voltage supply connected to a piezoelectric transducer (piezoelectric inkjet printing), or (2) increasing the temperature of the formulation (to slightly higher than its boiling point) which leads to thermal inkjet printing [49,50].

Piezoelectric (piezo) inkjet printing technology is the most acknowledged industrial inkjet printing process. In this approach, a piezoelectric crystal (ceramic actuator) undergoes distortion by the effect of an electric field that creates a pressure pulse in the ink chamber forcing drops to eject from the nozzle. The droplet size is correlated with the nozzle dimensions [51].

Combination of MNs (as drug delivery systems) with piezoelectric inkjet printing (as precise drug coating technology) permits an advanced approach in the pharmaceutical arena. Boehm *et al.*, fabricated biodegradable polyglycolic acid MNs coated with voriconazole (antifungal agent) using piezoelectric inkjet printing. This system was compared with unmodified and vehicle modified MNs against different micro-organisms (*Candida albicans*, *Escherichia coli*, *Pseudomonas aeruginosa* and *Staphylococcus aureus*). Voriconazole-polyglycolic acid MNs showed antifungal activity against *Candida albicans* while other devices were ineffective. The method was identified as a useful application of piezoelectric inkjet printing for drug loading onto MNs for poorly soluble pharmacological agents [52]. Micronazole (antifungal agent) was also printed onto MNs created from Gantrez^®^ AN 169 BF (poly(methyl vinyl ether-*co*-maleic anhydride)) using piezoelectric inkjet printing technology. Dimethyl sulfoxide was used as a solvent to enhance the antifungal drug penetration. Miconazole-loaded Gantrez^®^ AN 169 BF MNs exhibited antifungal activity against *Candida albicans* [50].

Three anticancer agents with varying solubilities (5-fluororacil (5-FU), curcumin (CRC) and cisplatin (CPT)) were utilized for transdermal delivery using MNs. At various drug-polymer ratios, anticancer agents with soluplus^®^ coatings (a copolymer used to increase the solubility of water insoluble drugs, hence dissolution rates) were uniform, reproducible and printable onto metallic MNs using the piezoelectric inkjet printing approach. The release profile depended on drug solubility. Hydrophilic 5-FU showed a rapid release profile compared to water insoluble CRC and CPT. However, varying antiproliferative action was observed for the three anticancer agents. Antiproliferative activity was concentration dependent, and at low concentrations (15 µg/mL) no antiproliferative activity was observed but observation increased with drug dose. This was dependent on drug potency as 7% and 9.4% viability was observed for 7 µg/mL (CRC) and 200µg/ml (CPT), respectively, sufficient to trigger antiproliferative activity. 5-FU was least potent with 20% cell viability at 400 µg/mL [51].

Piezoelectric inkjet printing technology was also combined with visible light dynamic mask micro-stereolithography-micromolding as a new approach of engineering polymeric MNs. Beohm *et al.*, used this combination of two prototyping methods to fabricate biodegradable acid Gantrez AN-139 anhydride copolymer MNs containing quantum dots. Deposition of quantum dots was confirmed in deeper layers of the skin (>200 μm depth from surface) after successful penetration into the stratum corneum. This valuable combination enables the development of sensors and theranostic devices (combination of detecting and drug delivery in one system) [53].

**Table 1:** Summary of selected MN coating techniques used with research details in selected studies.

Coating Method	Base MN Material	MN Type	Coating Material Type	Excipients	Active or Model	Coating Structure on MN	Points on Process	Comments	Ref.
**Dip Coating**	Stainless Steel	Flat. 700 µm in length	Molten solutions	PEG	Lidocaine	Film	Two main steps (dipping and drying). Additional time required for the preparation of formulation in hot-stage (including mixing) and further mixing using sonication	Lidocaine-PEG coated MNs had significantly higher delivery of drug (in 3 min) as compared with the topical administration of 0.15 g EMLA^®^. Method can be considered for hydrophobic drugs	[31]
	Titanium	340 µm in length	Solutions	Sucrose Polysorbate 20	rhGH	Film	Two main steps (dipping and drying). Roller drum method used to coat MN tips which were optimised to allow coatings to dry efficiently (5 s) before next dip. Ambient temperature process	Uniform MN coating achieved using high concentration of rhGH. Administered using an applicator. MN tips coated with formulation	[32]
	Stainless Steel	Single MNs, in-plane rows of MNs and out-of-plane arrays of MNs Flat.	Solutions and Particles	CMC Sodium salt and Lutrol F-68 NF	Vitamin B, Calcein, gWiz™ luciferase plasmid DNA, Sulforhodamine, BSA, BaSO_4_ particles and modified Vaccinia Virus	Film and Particles	Two steps (dipping and drying). Modified dipping process using horizontal axis. Process required micro-positioning device to allow MN coating through precision holes which overcomes meniscus rising and subsequent unwanted spreading. Formulation fed into a 2-plate system allowing MNs to be coated. Method monitored in real time through stereo microscope visualisation	The coated materials on the MNs shafts dissolved within 20 s in porcine cadaver skin with complete delivery into the skin. Precision coating and reduced wastage of material due to two plate coating system	[13]
**Gas-jet Drying**	Silicon	60 and 90 µm in length, Cone	Solutions	MC, Quil A, Poloxamer	OVA protein vaccine/FLR-dye	Film	Two step process. Includes the application of formulation and then drying based on gas-jet with variable speeds at specific incident angles	Densely packed MN successfully coated using this method. Method can be considered for large molecules.	[17]
	Silicon	110 µm in length, Segments	Solutions	MC, Trehalose and _14_C-OVA	Human Influenza Vaccine (Fluvax^®^)	Film	As above. MN patches were rotated to ensure uniformity. A nitrogen gas-jet was used	An improved approach to deliver vaccine to low-resource regions with long time stability. Tracer was incorporated into coating	[35]
**Spray Coating**	Silicon	280 µm in length, Contour	Solutions	HPMC, CMC, Tween 80		Film	Multiple variables can be used for spray optimisation. Coated MNs were dried for 12 h at the ambient temperature. Factorial design used to determine best coating formulation	Various conventional tablet coating polymers deployed for coating MNs. Multiple variables involved which impact spraying time. Surfactant may be required to improve coalescence of droplets	[36]
	Silicon	300 µm in length, Contour	Solutions	CMC, Trehalose, Maltodextrin, Sodium salt, Tween 80 and Lutrol F68	rADV, modified MVA Vectors and FITC	Relics and Films	Process optimised to control direct deposition on to MNs. This also required careful isolation of viruses during deposition. Multiple variables can be used for spray optimisation. Coated MNs were dried under vacuum (with desiccant) for a further 2–24 h	Uniform coating significantly preserved the virus’s activity which was successfully delivered into the skin and resulted in antibody response equivalent to the response induced by transdermal injection of the same vaccine	[37]
**EHDA Process**	Stainless Steel	500 µm in length, Flat	Solutions	PVP	FLR dye	Particles and Fibres	Multiple variables in this process. Reduced drying time due to non-aqueous solvent deployment for formulation. Coating thickness variable—dependent on deposition time. Ambient condition process	Solution properties used to prepare coating formulations are critical to the process and lead to variations in coating structure type	[34]
**Ink-jet Printing**	PMVE-MA	~800 µm in length	Solutions	DMSO	MNZ	Micro-droplet Film	MNs were exposed to UV light prior to printing with formulation. Ambient temperature process. Six layers of printed patterns applied. 38 µg of MNZ dose per patch prepared	Printing system presents an opportunity for poorly soluble anti-fungal drugs. A multi-mode engineering approach is a valuable for drop on demand system coatings	[50]
	Stainless Steel	700 µm in length, Flat	Solutions	De-ionised Water, Ethanol and Soluplus	5-FU, Curcumin, Cisplatin and Na FLR	Spotted and Micro- droplet Film.	Plotting of droplets on to MNs at 45°. Droplets deposited in continuous jetting cycles to increase coating. Process is computer controlled to determine volumes and real time deposition via imaging	Controlled deposition (of a droplet) using a controlled deposition device. Piezo-electric jet head used. Droplet size correlates with nozzle exit	[51]
	PGA	~800 µm in length, Half conical	Solutions	PMVE-MA, DMSO	VNZ and Methylene blue	Micro-droplet Film	Small quantities of formulations loaded into printer cartridge. 1 µg of the drug onto each MN patch system. Precision controlled deposition. Three layers deposited	VNZ-PGA MNs showed antifungal activity against *Candida albicans*. Accordingly, this system is ideal for poorly soluble pharmacological agents	[52]

**MC**: Methyl cellulose; **HPMC**: Hydroxypropylmethylcellulose; **CMC**: *Carboxymethyl cellulose*; **BSA**: Bovine serum albumin; **PEG**: Polyethylene glycol; **PMVE/MA**: Poly(methyl vinyl ether-*co*-maleic anhydride; **PVP**: Polyvinylpyrrolidone; **PGA**:polyglycolic acid; **FITC**: fluorescein isothiocyanate; **FLR**: Flourescein; **_14_C-OVA**: 14-C Ovalbumin; **OVA**: Ovalbumin; **VNZ**: voriconazole; **MNZ**: Miconazole; **5-FU**: Fluorouracil; **rADV**: recombinant human adenovirus; **rhGH**: recombinant human growth hormone; **MVA**: vaccinia virus Ankara; **DMSO**: dimethyl sulfoxide.

## 4. Conclusions

Microneedle based delivery of therapeutics is a rapidly evolving area in the pharmaceutical remit, and within the approach of microneedle based delivery, several sub-classes exist. Coated MNs offer advantages over existing routes of parenteral drug delivery including pain-free and self-administration options (when compared to injections). Coated MN systems also possess benefits over other MN types, including the ability to pattern MN surfaces and control the dose (based on the coating formulation) in a facile manner. They also allow the retention of microstructures within the coated system and allow formulation to be located specifically onto MN templates. The coating of MNs can be achieved using several techniques, including emerging process such as EHDA technology, printing and gas jet drying. In particular, these current advances provide opportunities for developing advanced drug delivery systems for personalized and tailored transdermal medicines.
